# Prevalence of the Microsporidian *Nosema* spp. in Honey Bee Populations (*Apis mellifera*) in Some Ecological Regions of North Asia

**DOI:** 10.3390/vetsci7030111

**Published:** 2020-08-13

**Authors:** Nadezhda V. Ostroverkhova, Olga L. Konusova, Aksana N. Kucher, Tatyana N. Kireeva, Svetlana A. Rosseykina

**Affiliations:** 1 Invertebrate Zoology Department, Biology Institute, National Research Tomsk State University, 36 Lenina Avenue, 634050 Tomsk, Russia; olga.konusova@mail.ru (O.L.K.); kucheraksana@gmail.com (A.N.K.); emilia30@mail.ru (T.N.K.); rosseykina75@mail.ru (S.A.R.); 2 Department of Biology and Genetics, Siberian State Medical University, 2 Moskovsky Trakt, 634055 Tomsk, Russia

**Keywords:** honey bee, *Apis mellifera*, *Nosema ceranae*, *Nosema apis*, epidemiology, replacement, ecoregions, North Asia

## Abstract

Two species of microsporidia, *Nosema apis* and *Nosema ceranae*, are obligate intracellular parasites that are widespread in the world and cause the infectious disease (Nosemosis) of the Western honey bee *Apis mellifera*. Information on the prevalence and distribution of *Nosema* species in North Asia conditions is scarce. The main aim of the present study is to determine the prevalence of *Nosema* spp. (Nosemosis) in honey bees inhabiting some inland regions of North Asia (Western and Eastern Siberia, Altai Territory, Russia, and northeastern part of Kazakhstan). The objective of the paper is also to assess the influence of climatic factors on the spread of *N. ceranae*. Eighty apiaries in four ecological regions of North Asia (southern taiga, sub-taiga zone, forest steppe, and mountain taiga forests) were investigated with regard to distribution, prevalence, and diversity of *Nosema* infection in honey bees using duplex-PCR. *Nosema* infected bees were found in 65% apiaries of ecoregions studied, and coinfection was predominant (36.3% of *Nosema-*positive apiaries). Both *N. apis* and *N. ceranae* occur across subarctic and warm summer continental climates, but while *N. apis* predominates in the former, *N. ceranae* is more predominant in the latter. No statistically significant differences in *Nosema* distribution were identified in various climatic zones. In the sub-taiga zone (subarctic climate), low presence of colonies with pure *N. ceranae* and a significantly higher proportion of coinfection apiaries were revealed. Long-term epidemiological study of *Nosema* spp. prevalence in the sub-taiga zone showed a surprising percentage increase of *Nosema*-positive apiaries from 46.2% to 74.1% during 2012–2017. From 2012 to 2015, *N. apis* became a predominant species, but in 2016–2017, the coinfection was mainly detected. In conclusion, the results of this investigation showed that *N. ceranae* is widespread in all study ecoregions of North Asia where it exists in combination with the *N. apis*, but there is no replacement of *N. apis* by *N. ceranae* in the studied bee populations.

## 1. Introduction

In Western honey bees, two species of Microsporidia causing nosemosis have been described: *Nosema apis* Zander, 1909 [1] and *Nosema ceranae* Fries et al., 1996 [2]. The microsporidian *N. apis*, a relatively benign pathogen, was considered to be the only causative agent of nosemosis in *Apis mellifera* for a long time [3]. Since 2006, a second *Nosema* species, *N. ceranae*, was identified in *A. mellifera* populations worldwide [4,5,6,7,8,9,10,11,12,13]. It should be noted that *N. ceranae* had been described as a microsporidian parasite of the Eastern honey bee *Apis cerana* in 1996 [2]. The parasite *N. apis*, responsible for nosemosis type A, is characterized by moderate virulence (mainly nosemosis is accompanied by dysentery), and bee colonies can often cure themselves under favorable environmental conditions [3,6]. *N. ceranae*, responsible for nosemosis type C, has been associated with reduced honey production, weakness, and increased colony mortality not preceded by any visible symptoms [14,15,16]. *Nosema* species also differ morphologically, genetically, in their ability to adapt to temperature, survival, and effects on the host [17,18,19,20,21,22,23,24]. For *N. ceranae*, a relatively new parasite for the *A. mellifera*, data on the virulence, the prevalence in different climates, as well as the role and effect on the viability and survival of bee colonies are contradictory [14,19,25,26,27,28,29,30].

Conflicting results from different studies can be attributed to many factors, such as the biological characteristics of honey bees (caste, age of the bees, commercially and traditionally managed bees) [31,32], the genetic diversity of honey bees, bee subspecies and lineages [24,33,34,35], climatic and environmental differences [7,18,36], beekeeping practices [10], as well as diagnostic methods [37] and research conditions (number of analyzed bees, time and method of sampling, natural population research or experiment) [31,38,39]. *Nosema* species can only be confirmed using molecular methods [39], which can have different levels of resolution, for example, the single-copy *Hsp70* gene method qPCR detects a lower amount of *N. ceranae* copies compared to the multicopy *16S rRNA* gene method [37]. When conducting experimental infection studies, the laboratory results may be affected by specific conditions such as the duration of the experiment, the temperature and humidity, the method of infection, number of bees in the cages, the type of their diet, etc. [31,39].

To understand the mechanisms of *Nosema* disease and the effect of *N. ceranae* infection on the host it is necessary to study the prevalence and distribution of *Nosema* species, primarily *N. ceranae*, in different regions and climatic zones and the long-term dynamics of infection of honey bees with various *Nosema* species.

The prevalence of *Nosema* species in *A. mellifera* populations in Eurasia is currently studied in most European countries [6,25,40,41,42] and some countries of Southwest Asia as well [29,43,44,45,46,47]. As the sole causative agent of nosemosis, *N. ceranae* was detected in Croatia [48], Central Italy [49,50], Iran [43,47], and Saudi Arabia [45]. In South-European countries, such as Italy [6,49,50] and Greece [25], *N. ceranae* had indeed practically replaced *N. apis* while this was not observed in Northern Europe (Ireland, Sweden, Norway, and Germany) [30,38]. For example, in Sweden, in the spring of 2007, 89.0% of *Nosema*-positive bee colonies had *N. apis* and 10.0% colonies had mixed *N. apis/N. ceranae* infections [38]. On the contrary, in Scotland, 86.2% of *Nosema*-positive bee colonies had mixed infections [40]. In Germany, in 2009, three infection categories were widespread: 48.5% *Nosema*-positive bee colonies were with *N. apis*, 33.8% were infected with *N. ceranae*, and 17.6% had mixed infection [27]. 

Climate is considered to be one of the main factors in the spread of *Nosema* species [26,30,49]. In warmer climates, *N. ceranae* is more competitive than *N. apis*; in contrast, in cold climates, *N. ceranae* spores appear to be much more vulnerable than the *N. apis* spores [26]. Laboratory data also suggest that the spread of *N. ceranae* across the globe is reduced in colder climates as *N. ceranae* spores are capable of surviving high temperatures and desiccation but are intolerant of cold [17,18,27,36,51]. However, the impact of weather conditions on the distribution of microsporidian *Nosema*, primarily *N. ceranae*, in the field is poorly understood [26].

The different sensitivity to temperatures in the *Nosema* species may be a potential explanation for the wider *N. ceranae* prevalence in warmer (subtropical) climate (Southern Europe, for example, the Mediterranean countries) compared to *N. apis*, which is more prevalent in temperate climates (Northern countries) [26]. Today, bees are threatened, and the cause of the problem is still unknown, which is why it is being described as Colony Collapse Disorder (CCD). Researchers suspect this may be due to a combination of various diseases including *N. ceranae*, environmental pollution, and farming practices, mainly due to large monoculture cropping and toxic phytosanitary products [52,53,54]. For the first time in Spain, bee colony mortality was clearly attributed to *N. ceranae* infection [7,14,15,16,55,56]. In 2004–2006, the prevalence of *N. ceranae* in dead bee colonies was about 90% [7]. It is assumed that colony collapse caused by *N. ceranae* is not restricted to Spain but is a global problem, at least it is regarded as a Europe-wide phenomenon. For example, in some Mediterranean countries, such as Greece [25], Israel [29], and Turkey [44,46], bee colony losses are also associated with *N. ceranae* infections. However, in Northern Europe, colony collapse could not be associated with *N. ceranae* [27,57]. These data pointed to climatic factors differentially influencing the prevalence and the virulence of *N. ceranae* in Europe [26,27,28] and/or differences in *N. ceranae* susceptibility between regionally predominating *A. mellifera* subspecies [24,34,39].

The prevalence of *N. ceranae* in more severe conditions in the continental regions of North Asia has not been adequately studied yet [13,58,59]. The purpose of this study was to determine the prevalence of *Nosema* infection in honey bees inhabiting some ecological regions of North Asia (southern taiga, sub-taiga zone, forest steppe taiga, and mountain taiga forests) and assess climatic factors that influence prevalence.

## 2. Materials and Methods 

### 2.1. Ecological and Geographical Characteristics of the Region

**Figure 1:**
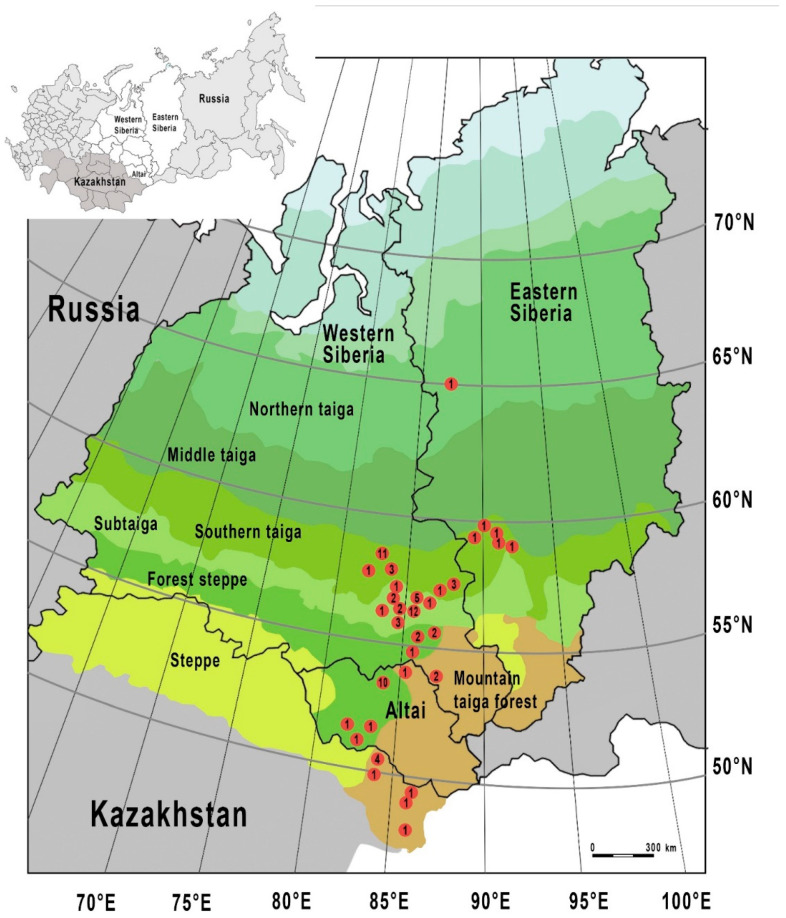
Distribution of monitored apiaries within the study ecoregions of North Asia (southern taiga, sub-taiga zone, forest steppe taiga, and mountain taiga forests). The number of the examined apiaries in the locality is indicated by numbers.

We explored the *Nosema* infestation of honey bees in several regions of North Asia (Western and Eastern Siberia, Altai Territory, Russia, and northeastern part of Kazakhstan). The study apiaries were located in an area extending from 48° N to 65° N in the meridional direction and from 81° E to 92° E in the zonal direction (Figure 1).

In this region, located at a significant distance from the ocean, the climate is regarded as a continental one (Köppen climate classification). The characteristic features of the continental climate are cold winters and hot summers. Air masses are usually cold in winter, warm in summer, but always relatively dry continental. The average temperature is above 10 °C in the warmest months, and during the coldest month, it is on the average below 0 °C. The annual temperature amplitudes can be 70 °C. The vegetation period in the regions of North Asia is short, wintering of honey bees is long (it usually lasts for 6 months, but sometimes it is up seven months).

In North Asia, various ecological regions are represented (Figure 1). We investigated apiaries located in 4 ecoregions, namely southern taiga, sub-taiga zone, forest steppe taiga, and mountain taiga forests (Table 1). In these regions, beekeeping is well-developed, especially there are lots of apiaries in the sub-taiga zone, forest steppe taiga, and mountain taiga forests. In the southern taiga, there are many apiaries, but they are located in far-away areas. In addition, we also investigated one apiary (near Turukhansk, 65°47′35″ N, 87°57′44″ E) located in the Northern taiga (the north of Eastern Siberia; Figure 1). Beekeeping is not well-developed in this ecoregion due to a very cold climate, but there exist single apiaries in that area [59].

Selected apiaries were situated in both flatland (southern taiga, sub-taiga zone, forest steppe taiga) and mountainous (mountain taiga forests) parts under different climatic conditions. The southern taiga and sub-taiga are characterized by a subarctic climate, while the forest steppe taiga and mountain taiga forests are distinguished by a warm summer continental climate (Table 1). Although the southern taiga and sub-taiga are characterized by a subarctic climate, there are some differences in the average monthly and average annual temperatures, sum of active temperatures above +10.0 °C (Σt). For the southern taiga zone, significant variability of these indicators is observed. Similarly, for zones with a warm summer continental climate (forest steppe taiga and mountain taiga forests), a significant range of the main climatic indicators is surveyed in the zone of mountain taiga forests (Table 1).

### 2.2. Historical Background

In Siberia and Altai Territory, the honey bee was introduced about 230 years ago. It was a dark forest bee *Apis mellifera mellifera* that well-adapted to the local climate and plant communities as well. The honey bee population is an artificial population whose wintering is controlled by people [60]. The first apiaries were formed in the mountainous regions of Western Siberia and Altai Territory. Subsequently, the development of beekeeping took place in Eastern Siberia, namely in the southern taiga, sub-taiga, and forest steppe.

### 2.3. Research Algorithm

It should be noted that we have conducted our research according to two lines. The first line of research is the study of *Nosema* infection of honey bees in apiaries in North Asia. A total of 80 distant apiaries located in four ecoregions (southern taiga, sub-taiga zone, forest steppe taiga, and mountain taiga forests) were monitored for *Nosema* infection between spring 2016 and autumn 2017 (for most apiaries, the material was mainly collected in the early summer due to the long cold spring; usually after the first flight of bees). We examined 24 apiaries in the southern taiga, 27 apiaries in sub-taiga, 18 apiaries in forest steppe taiga, and 11 apiaries in the mountain taiga forests (Figure 1). In addition, in the northern taiga, one apiary near Turukhansk was also investigated. Within each apiary, a minimum of three colonies (10% of the total colonies) were randomly selected with regard to *Nosema* detection.

The second line of research is connected with a retrospective analysis (a long-term dynamics) of *Nosema* infestation of apiaries in the sub-taiga zone in 2012–2017. A total of 79 distant apiaries were analyzed. For a retrospective study, we used bee samples collected in sub-taiga apiaries from 2012 to 2017 and stored them in a biobank at a temperature of −20 °C.

It is worth pointing out that the apiaries were visited only once. In each sampling, forager bees were collected at the entrance of each sampled hive. For each bee sample, the beekeeper provided information, including the location, history and other characteristics of the apiary, the origin of bees, the incidence of bee colonies, etc. No hives of the apiaries sampled had a history of external signs referable to nosemosis and no signs of the disease were present at the time of sampling. Mass death of bees after wintering in the study apiaries was not observed. Between 60 to 70 workers from each bee colony were pooled and used for DNA isolation. The presence of nosemosis in the bee colony was examined using a polymerase chain reaction (PCR).

### 2.4. Experimental Procedures

DNA was extracted from the midgut of bees (a pool of 60–70 individuals was formed) using a DNA purification kit, PureLink™ Mini (Invitrogen, Carlsbad, CA, USA) according to the manufacturer’s protocol. After the DNA extraction, the samples were submitted to duplex-PCR [7]. For the diagnosis of two *Nosema* species, we used two types of primers. The primer sequences used to amplify the 321 bp fragment corresponding to the 16S ribosomal gene of *N. apis* were 321APIS-FOR 5′-GGGGGCATGTCTTTGACGTACTATGTA-3′ and 321APIS-REV 5′- GGGGGGCGTTTAAAATGTGAAACAACTATG-3′. The primer sequences utilized to amplify the 218 bp fragment corresponding to the 16S ribosomal gene of *N. ceranae* were 218MITOC-FOR 5′-CGGCGACGATGTGATATGAAAATATTAA-3′ and 218MITOC-REV 5′-CCCGGTCATTCTCAAACAAAAAACCG-3′ [7].

PCR was performed using a thermal MyCycler T100 (BioRad, Foster City, CA, USA) in a reaction volume of 20 μL containing 1 μL of template DNA, 1× PCR buffer, 1.5 mM MgCl_2_, 200 μM of each dNTP, 0.2 μM of each forward and reverse primer and 1U Taq polymerase (Fermentas, Thermo Fisher Scientific, Chelmsford, MA, USA). The routine consisted of an initial denaturation step at 94 °C for 2 min, followed by 35 cycles of 94 °C for 30 s, 58 °C for 30 s and 72 °C for 1 min, and a final extension step at 72 °C for 5 min. PCR products were analyzed on 1.5% (*m*/*v*) agarose gels. Gels were stained with ethidium bromide and visualized using UV illumination (Gel Doc XR+, BioRad, Foster City, CA, USA). All analyses were carried out in duplicate and identical results were obtained. For each PCR, positive control (reference *N. apis* and *N. ceranae* DNA extracts as template) was used. Negative control (ddH_2_O) was also included in each run of PCR amplification to detect possible contamination.

If only *N. ceranae* or only *N. apis* were detected in all examined bees, the apiary infection category was pure *N. ceranae* or pure *N. apis*, respectively. An apiary was considered coinfected if both *Nosema* species were detected in honey bees.

### 2.5. Meteorological Data

To study the climatic characteristics of ecoregions (Table 1), we used reference material [61,62,63] including the average indicators of meteorological observations over a long-term period (for 30 years), namely, the average annual temperature and average annual precipitation, the average temperature in January and July, the duration of the frost-free period and the sum of active temperatures (Σt).

To assess long-term temporal trends in the prevalence of two *Nosema* species in the sub-taiga zone and analyze the associations of the prevalence of *Nosema* infection with climatic factors, we used the meteorological data obtained by the Tomsk weather station, 56°29′ N, 84°56′ E [62]. The following data were employed: average annual temperatures, average daily temperatures, duration of the period with active temperatures, sum of precipitation just for the same period of the year. Reference data were used to calculate two parameters: sums of active temperatures (Σt) and hydrothermal coefficient (HTC).

The sum of active temperatures is one of the main indicators of the territory’s thermal resources. Active temperatures designate daily average temperatures above +10.0 °C. The sum of the active temperatures (Σt) is calculated for a period with an average daily air temperature above +10.0 °C.

To characterize the moisture supply (humification conditions) in the area concerned, we used the hydrothermal coefficient suggested by Selyaninov (HTC) [64]. The hydrothermal coefficient (K) is calculated as the ratio of precipitation (R) to the sum of temperatures (Σt) for a period with temperatures above +10.0 °C: K = R*10/Σt. At HTC values 1.1–1.4, moisture supply is regarded as optimal; at values 0.76–1.0 it is insufficient, and at values 1.41–1.5 it is an elevated one.

### 2.6. Statistical Analysis

The results of the research are presented using numbers and percentages. Statistical significance of qualitative data was determined using the Chi-square test or the Z-test for proportions. To compare apiaries infected and uninfected with *Nosema* between different ecoregions, we used a two-sample proportion Z-test. The Chi-square test was used to compare the incidence of *Nosema* infection in the sub-taiga zone between three sampling years. In the case of a small number of one of the comparison classes, we used the chi-square test with Yates’ correction. *Nosema*-positive, *N. apis*-positive, *N. ceranae*-positive, and coinfected apiaries were subgroups to be compared. Statistical analysis was performed using the Statistica program.

## 3. Results

### 3.1. Infestation of Apiaries with Nosema Species in Four Ecoregions of North Asia in 2016–2017

Honey bee samples collected in 2016–2017 from 80 apiaries of several ecological regions of North Asia were examined for the presence of *Nosema* infections (Table 2). In all the ecoregions of North Asia studied, two species of Microsporidia were registered in honey bees: *N. apis* and *N. ceranae*. In total, among 80 apiaries which had been analyzed, *Nosema* spp. were detected in 52 honey bee apiaries (65.0%): 15.0% of *Nosema*-positive apiaries were infected with only *N. apis*; 13.75%—only *N. ceranae*; in most apiaries (36.25%), both *Nosema* species were detected. In general, in North Asia, the infestation of apiaries with both types of *Nosema* (coinfection) was higher compared to the infestation of apiaries with only *N. apis* or only *N. ceranae*, but statistically significant differences were not shown (proportion Z-test; Z = 1.31, *p* > 0.05).

**Figure 2:**
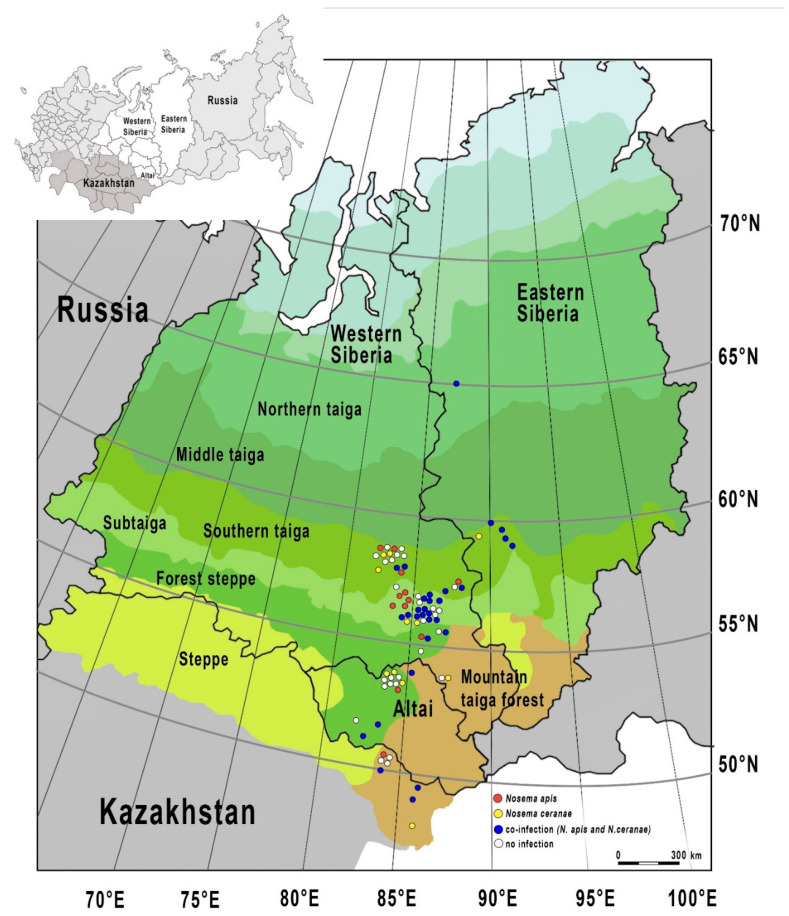
Prevalence of *Nosema* infections in several ecoregions of North Asia (southern taiga, sub-taiga, forest steppe, and mountain taiga forests).

In all study ecoregions of North Asia (southern taiga, sub-taiga, forest steppe, and mountain taiga forests), a significant number of apiaries infected with *Nosema* were detected: ranging from 50.0% of apiaries in the forest steppe zone to 74.1% in the sub-taiga zone (Figure 2).

At the same time, the distribution of *Nosema* species differed in the study ecoregions (Table 2). We detected three possible infection categories of apiaries: only *N. apis* infection, only *N. ceranae* infection, and coinfection of two *Nosema* species. coinfection prevailed in apiaries of all the ecoregions and it was detected in 65.0% of *Nosema-*positive apiaries (Figure 2).

The spread of *Nosema* infection in apiaries of various ecoregions was as follows:

(i) In the southern taiga, the number of apiaries infected with either *N. apis* or *N. ceranae* was the same (16.7%).
(ii) In the forest steppe and mountain taiga forests (similar climatic conditions—warm summer continental climate), the spread of *Nosema* species is similar: 9.1% and 11.1% of apiaries with only *N. apis*; 16.7% and 18.2% of apiaries with only *N. ceranae*; 22.2% and 36.4% of apiaries with coinfection (*N. apis* and *N. ceranae*), respectively (proportion Z-test; Z < 0.39, *p* > 0.05).
(iii) In the sub-taiga zone, the spread of Nosema infection in apiaries is different from that in the other ecoregions. The smallest number of apiaries with only *N. ceranae* (7.4%) and the highest number of apiaries with mixed infection (48.2%) were revealed. In addition, about a quarter of apiaries (18.5% of *Nosema*-positive apiaries) are infected only with *N. apis* (similar to the southern taiga zone; subarctic climate).

However, no statistically significant differences in the distribution of both common *Nosema* infections and individual *Nosema* infection (pure *N. apis*, pure *N. ceranae*, coinfection) were found between the ecological regions (Z < 1.76, *p* > 0.05). Additionally, no statistically significant differences in *Nosema* distribution were identified in various climatic zones (subarctic climate and warm summer continental climate; Z < 1.52, *p* > 0.05).

### 3.2. Dynamics of Infection of Apiaries with Different Nosema Species in Sub-Taiga from 2012 to 2017

Despite the fact that no significant differences were found in the incidence of *Nosema* infection both between ecological regions and climates (probably due to the small number of study apiaries), there exist some trends in research. For example, a difference in *Nosema* prevalence characteristic of the two groups of apiaries with coinfection between the sub-taiga and forest steppe zones approached the level of statistical significance (proportion Z-test; Z = 1.76, *p*  <  0.10).

To gain a more complete understanding of the spread of *Nosema* infections in the sub-taiga zone, we examined long-term temporal trends in the prevalence of two *Nosema* species from 2012 to 2017 (Table 3).

In the course of six years from 2012 to 2017, there was a surprising increase in *Nosema* infection in the sub-taiga zone (Table 3). The percentage of *Nosema-*positive apiaries increased from 46.1 to 57.7% over a period of the first four years to 74.1% during the last two years (χ^2^ = 4.316, *p* = 0.038). If in 2012–2015, *N. apis* was predominant among the other infectious categories compared to 2016–2017 (χ^2^ = 10.800, *p* = 0.002) whereas, in 2016–2017, coinfection (*N. apis* and *N. ceranae*) was mainly detected among *Nosema*-positive groups (χ^2^ = 5.298, *p* = 0.022; Table 3).

Three various periods with regard to the frequency of coinfection apiaries were established in the following order: from 2012 to 2013, from 2014 to 2015, and from 2016 to 2017. During the first period (2012–2013), no apiary with coinfection was detected. In the course of the second period (2014–2015), a small number of apiaries with coinfection was found (11.5%). Interestingly, the number of apiaries, either with only *N. apis* (42.3%), or with only *N. ceranae* (3.9%), was the same during these study periods (2012–2013, and 2014–2015). Finally, in the course of the third period (2016–2017), coinfection was identified in about half of the study apiaries (48.2%), which is statistically different from the 2014–2015 period (χ^2^ = 6.776, *p* = 0.01). In addition, the number of apiaries where only *N. apis* was diagnosed decreased by half (from 42.3 to 18.5%; χ^2^ = 3.557, *p* = 0.06). In 2016–2017, the smallest number of apiaries was detected with only *N. ceranae* (7.4%) but it doubled during the observation period, from 3.9% to 7.4% (χ^2^ = 0.001, *p* = 0.97).

We tried to assess the influence of both climatic factors separately (temperature, humidity) and the integrated indicator “hydrothermal coefficient” (HTC) on the spread of *Nosema* infection in the sub-taiga zone in 2012–2017.

An increase in the *Nosema* infection of honey bees and the spread of *N. ceranae* in the sub-taiga zone may be associated with an increase in the duration and heat supply of the active life period of biological objects from 2012 to 2017 (Table 4). On the whole, the indicators of the temperature regime in the sub-taiga zone from 2012 to 2017 reflect a general tendency of warming of a landing air layer in the Russian territory. So, average annual temperature deviations from average long-term values toward an increase by 1.2–3.1 °C were observed in the study area. The biggest deviations were identified in 2013 and from 2015 to 2017. What is more, abnormally high indicators of the vegetation period heat supply were observed in 2012 and in 2015–2017. For example, in 2016, the sum of active temperatures (Σt) comprised 2217 degree days in view of a basic value of 1650–1800 degree days.

We also observe significant variations in such climatic indicators as the period with active temperatures and the hydrothermal coefficient (HTC) in the study area (Table 4). For example, in 2012–2017, the hydrothermal coefficient (HTC) ranged from 0.92 to 1.49. An alternating number of years with an insufficient period of moisture supply (2012, 2014, 2016) was followed by a period of optimal moisture supply. It is interesting to note that the moisture period of 2017 is characterized as an excessive one.

The year 2012 along presents a certain interest because it was characterized by high summer temperatures and a low moisture supply. During the active growing season, 86 cases of the absolute maximum air temperature exceeding in the course of one day were recorded. Most cases were reported in the south of Western Siberia, where abnormally hot weather was observed. For example, in the Tomsk Region (Western Siberia), a significant deviation temperature from the required standard (from 1.3 °C to 7.2 °C) was first observed in June–July over the past 60 years. In Tomsk in particular (56°29′ N, 84°56′ E), the temperature deviation from the average long-term standard came to 5.7 °C in June and 5.3 °C in July. According to the data of the Tomsk weather station, in June, the amount of precipitation was 54% according to the required standard, it comprised only 33% of the standard data. In Tomsk, in July, the HTC value accounted for 0.35, which was indicative of severe drought. That is the reason for the extreme meteorological conditions of 2012 which were quite different from those during a long-term observation period in this area [65].

Under these conditions, we expected a widespread occurrence of Microsporidia, primarily *N. ceranae*, in this region. However, in the 2012 beekeeping season, we did not find a single bee colony infected with *Nosema* spores, and the bees were distinguished by a high flying activity and a high honey productivity as well. Therefore, there is no need for making any assumptions relating to the impact of climatic conditions on the *Nosema* distribution in 2012. It is probably not the temperature, but the good resistance of the examined bee colonies to Microsporidia which is the determining factor.

In 2013–2014, the heat supply to the growing season was very low (86 and 90, respectively), whereas, in 2015–2017, the period with active temperatures was the longest (from 124 to 128 days). If in 2013–2015, in apiaries of the sub-taiga, *N. apis* was the predominant species, then in 2016–2017, coinfection was most common. None of the studied climatic indicators can be associated with the spread of *Nosema*, coinfection in particular, in the sub-taiga zone.

Probably, both biotic and abiotic factors are involved to a greater or lesser extent individually or synergistically. Environmental conditions can exert a direct impact on the parasite or indirectly influence them by altering host physiology, behavior, and immunity [36].

## 4. Discussion

We have investigated the spread of *Nosema* species in several ecological regions of North Asia (southern taiga, sub-taiga, forest steppe, and mountain taiga forests) and the influence of climatic conditions (subarctic and warm summer continental climates) on the prevalence of *N. ceranae*.

The geographic distribution of *Nosema* spp. in several ecoregions of North Asia can be represented as follows:

(i) The presence of bee colonies infected with pure *N. ceranae* in all the study ecoregions;
(ii) No significant differences were identified in the incidence of *Nosema* infection between subarctic and warm summer continental climates. There is only a trend towards a high proportion of apiaries with only *N. apis* infection in the subarctic climate, and vice versa, towards a higher proportion of apiaries with only *N. ceranae* infection in warm summer continental climate;
(iii) Coinfection detected in most study apiaries of North Asia;
(iv) A higher proportion of coinfection apiaries and a lower presence of colonies with pure *N. ceranae* in the sub-taiga zone (subarctic climate);
(v) There is no replacement of *N. apis* by *N. ceranae* in the study honey bee populations of North Asia, but their coexistence is registered.

Our results show the widespread use of *N. ceranae* in all study ecoregions of North Asia, as has been documented in many regions of the world [66]. In addition, *N. ceranae* was found in the apiary in very cold climates in Northern taiga (near Turukhansk). Our epidemiology data clearly show that *N. ceranae* became successfully established and expanded its presence in honey bee populations in both the southern and northern regions of North Asia. It seems likely that changes in the spread of two *Nosema* species should be considered in the context of stable changes to the heat supply regime of the study area. Moreover, the spread of the parasite during a certain period is determined by the weather conditions of the previous period. For example, unfavorable conditions in 2013–2014 could have been the reason for a decrease in bee resistance to diseases and, consequently, for the spread of infection in subsequent years (2015–2017), characterized by an increase in heat supply during the growing season.

The increasing worldwide prevalence of *N. ceranae* in the past decade, and, conversely, a decrease in the *N. apis* prevalence (even the absence of this parasite) in some regions suggests that *N. ceranae* might be displacing *N. apis* [6,26,67]. However, while in Southern Europe, especially in the Mediterranean countries like Spain, Italy, Israel, Greece, and Turkey, *N. ceranae* has been the dominant species for 10 years [6,25,37,49], in Northern Europe (Ireland, Sweden, Norway, and Germany), *N. apis* is still the predominant species [27,30,38]. For example, in Sweden, the majority of bee colonies (89%) were infected only with *N. apis*, and in other bee colonies, coinfection was identified [38]. In Europe, there is a South to North gradient in the distribution of *Nosema* species, which can be determined by the climatic characteristics of the regions [6,27,30]. In European countries with hot summers and moderate winters, *N. ceranae* is predominant and nearly replaced *N. apis* over the past decade in Spain and Italy [5,6,7,14]. On the contrary, in countries with rather cold and long winters such as Sweden and Germany, *N. apis* is a prevailing species [27,30]. For example, in Northeast Germany, despite a significant increase in *N. ceranae* prevalence during the last 12 years, no replacement of *N. apis* by *N. ceranae* took place in the honey bee population. For replacement of *N. apis* by *N. ceranae* at the population level, a simple increase in *N. ceranae* infection prevalence is not sufficient but *N. apis* infection prevalence should have concomitantly decreased during the study period. However, in Northeast Germany, a significant decrease in *N. apis* infection prevalence was only observed in autumn, and no significant change in *N. apis* infection prevalence was found in spring [30].

In this study (for example, sub-taiga zona) and in our earlier studies of various regions of North Asia, for example the Tomsk Region [13], we also showed that starting from a very low level, the prevalence of *N. ceranae* infections significantly increased continuously in the observed honey bee populations over the last six years. However, unlike Northeast Germany, where values for coinfection prevalence of bee colonies ranged between 0.0% and 10.0% during 2005–2016 [30], in North Asia, coinfection is widespread and was found in more than 30% of apiaries. However, a similar distribution pattern of *Nosema* infection has been identified in North Asia and Scotland. So, in Scotland, in the 70.4% of the bee colonies, the presence of both *N. ceranae* and *N. apis* was detected [40]. Interestingly, an increasing gradient of coinfection from North to South was also observed in some countries of the southern hemisphere, for example, in Argentina: coinfection was more prevalent in regions with temperate (77.9%) as compared to those of subtropical climate (22.1%) [68].

The high prevalence of coinfection (*N. ceranae* and *N. apis*) in the studied ecoregions of North Asia may suggest that *N. ceranae* is moving from warm summer continental to subarctic climate regions. Despite the distribution of *N. ceranae* infection in more severe climatic conditions (subarctic climate), we revealed some trends in the prevalence of pure *Nosema* infections in subarctic and warm summer continental climates. In warm summer continental climate, pure *N. ceranae* infection predominates, while in subarctic climate, pure *N. apis* infection is widespread.

As recently demonstrated in laboratory studies, mixed infections (*N. apis* and *N. ceranae*) negatively affected honey bee survival more than single *Nosema* infections [69]. However, contrary to this publication, in Siberia, bee colonies living in nature in very cold climates for a long time were relatively healthy, and *N. ceranae* is not associated with colony depopulation or honey bee collapse.

To determine the possible causes of the observed pattern of *Nosema* infection in honey bees in North Asia, we studied the history of beekeeping in Siberia and Altai Territory, namely, bee diseases and registered cases of mass death of bee colonies. A characteristic feature of the honeybee populations in Siberia is their long-term habitation in an isolated area. Honey bees have adapted to very cold climates. Under these conditions, certain parasite–host relationships, including Microsporidia, were formed.

Previously, nosemosis in honey bees in Siberia and Altai Territory was attributed exclusively to *N. apis*. The first description of *N. ceranae* infection in bees in North Asia using molecular genetic methods refers to 2009: the Tyumen Region, Altai Territory [58], and the Tomsk Region [13,60]. In addition, we identify the *N. ceranae* in the *A. m. mellifera* bee colonies from the long-isolated apiaries in the far-away taiga (Yenisei population of Krasnoyarsk Krai, Eastern Siberia), where new bees have not been imported for more than 60 years [59]. Our results provide evidence that *N. ceranae* infection occurs in bee colonies living in cold climates in Siberia, and this parasite is not associated with a colony depopulation or honey bee collapse. The connection of a long wintering and the nosemosis caused by *N. apis* was noted, but cases of mass bee mortality in study areas of North Asia are rather rare [70,71].

The first case of mass mortality of bee colonies in Siberia from the southern taiga to the forest steppe and mountain forests was described in the 1880s. However, the reasons for such an occurrence remain unknown. The first documented cases of mass bee mortality from nosemosis date back to 1914–1917 [71]. The presence of the causative agent was confirmed both by the clinical picture and by the microcopy of the pathological material. It is interesting to note that in the following years (1980s), the most noticeable losses of bee colonies in Siberia were associated with the rapid spread of varroosis (*Varroa destructor*) [70]. In 2017, in Altai Territory, the mass bee mortality was marked at the end of wintering [72]. The most probable reason for the spread of diseases, including nosemosis, and, possibly, the mass bee mortality is bee hybridization because the southern bee subspecies and hybrids characterized by a decrease in immunity did not withstand a particularly prolonged wintering. It can be assumed that diseases, including nosemosis, are probably a consequence of bee hybridization. The intensive importation of honey bees from the southern regions of Russia had been practiced in the Western Altai since the 1940s. For the southern taiga and sub-taiga this process is typical for the last two decades. Probably, both biotic and abiotic factors are involved to a greater or lesser extent individually or synergistically. Environmental conditions can exert a direct impact on the parasite or indirectly influence them by altering host physiology, behavior, and immunity [36]. In addition to the geographic and climatic factors, the genetic features of the host may affect the coadaptation of the honey bee and *Nosema* parasite, and the *Nosema* spp. prevalence in honey bee populations [24,34,68]. It is assumed that the variation between bee colonies in susceptibility to infection by *N. ceranae* is linked to genetic variability in workers from resistance to tolerance [24,34,39].

Thus, the issues relating to the distribution of *Nosema* spp. and the consequences of infection for bee colonies have not been resolved yet. The virulence of *N. ceranae* could be influenced by climatic conditions [23,26,28] or might actually depend on honeybee race, honeybee genetic diversity [33,34,73,74,75], and multiple *N. ceranae* genetic variants resulting in different biological consequences [76]. In this regard, it is of considerable interest to study the long-term and seasonal dynamics of *Nosema* infections in bee colonies, the relationship between pathogens and honeybees taking into account their genetic features, and the role of nosemosis pathogens in mass mortality of honeybees. Evidently, extended and detailed research is urgently required in order to elucidate a complete effect of *N. ceranae* infection on *A. mellifera* colonies in various geographical and climatic areas. The protective mechanisms behind this pattern remain to be studied.

## 5. Conclusions

The results of the research have shown that coinfection of two *Nosema* species, *N. apis* and *N. ceranae*, is widespread in all the ecological regions of North Asia studied. In the sub-taiga, a significant increase in *Nosema* infection has been observed for the last 6 years. There is no replacement of *N. apis* by *N. ceranae* in the studied bee populations. Despite the distribution of *N. ceranae* infection in subarctic climate, we have revealed some trends in the prevalence of pure *Nosema* infections in subarctic climate (pure *N. apis* predominates) and warm summer continental climate (pure *N. ceranae* is widespread). Further long-term research will contribute to understanding the interaction between the two *Nosema* species and the role of various factors, primarily climate, in the spread of these parasites of honey bee populations.

## Figures and Tables

**Table 1:** Geographic and climatic characteristics of the study ecoregions of North Asia.

Ecological Region	Area Coordinates	Climate (Group D) ^#^	Altitude, m	Average Temperature (°C)			Average Annual Precipitation, mm	Frost-Free Period (Days)	Σt, Degree Days *
				Annual	In January	In July			
Southern taiga (boreal forest)	from 57°00′00″–59°50′00″ to 82°39′00″–92°08′00″	Subarctic climate	60–130	−0.9–(−2.0)	−19.8–(−21.6)	17.9–18.3	250–500	90–120	1472–1820
Sub-taiga zone	from 56°15′00″–56°48′21″ to 83°58′00″–86°42′00″	Subarctic climates	90–120	−0.6	−19.2	18.1	350–550	100–120	1650–1800
Forest steppe	from 51°00′00″–55°19′59″ to 81°28′00″–85°30′00″	Warm summer continental climate	200–250	2.1–2.6	−16–(−19)	18–20	350–450	120–123	1900–2100
Mountain taiga forests	from 48°34′03″–53°45′00″ to 82°18′25″–87°07′00″	Warm summer continental climate	290–1685	0.3–2.4	−12.6–(−24.0)	17–22	500–900	123–135	2200–2400

^#^ Köppen climate classification was used. * Degree days are a specialist kind of weather data, calculated from readings of outside air temperature.

**Table 2:** Prevalence of colonies infected with only *N. apis* (*N. apis*) or only *N. ceranae* (*N. ceranae*) or coinfection (*N. apis* and *N. ceranae*) in apiaries in several ecoregions of North Asia from spring 2016 to autumn 2017.

Ecoregion	Total Number of Analyzed Apiaries	*Nosema* Infection not Detected		Infected Apiaries (Infection Categories)					
				*N. apis*		*N. ceranae*		*N. apis* and *N. ceranae*	
		N	%	N	%	N	%	N	%
Southern taiga	24	8	33.33	4	16.67	4	16.67	8	33.33
Sub-taiga zone	27	7	25.93	5	18.52	2	7.41	13	48.15
Forest steppe	18	9	50.00	2	11.11	3	16.67	4	22.22
Mountain taiga forests	11	4	36.36	1	9.10	2	18.18	4	36.36
Total	80	28	35.00	12	15.00	11	13.75	29	36.25

The total number of analyzed apiaries along with the numbers (n) and proportions (%) of apiaries within each infection category are presented.

**Table 3:** Spreading of *Nosema* species in apiaries of the sub-taiga zone from 2012 to 2017.

Study Period	Total Number of Analyzed Apiaries	*Nosema* Infection not Detected	Infected Apiaries (Infection Categories)		
			*N. apis*	*N. ceranae*	Coinfection
2012–2013	26	53.85	42.31	3.85	0
2014–2015	26	42.31	42.31	3.85	11.54
2016–2017	27	25.93	18.52	7.41	48.15
Total	79	40.51	34.18	5.06	20.25

**Table 4:** Temperature regime and humification conditions in the sub-taiga zone according to observations made at the Tomsk weather station, 56°29’ N, 84°56’ E.

Year of Study	Average Annual Temperature (°C)	Σt, Degree Days *	Period with Active Temperatures, Days	Amount of Precipitation for the Period with Active Temperatures, mm	Hydrothermal Coefficient (HTC)
2012	0.6	2064	121	199	0.96
2013	1.7	1587	86	200	1.26
2014	0.8	1590	90	162	1.01
2015	2.5	2107	128	277	1.31
2016	1.7	2217	128	203	0.92
2017	2.2	1912	124	285	1.49

* Degree days are a specialist kind of weather data, calculated from readings of outside air temperature. Σt—the sum of the temperatures for a period with an average daily air temperature above +10.0 °C. Active temperatures are average daily temperatures above +10.0 °C.
